# Effectiveness of continuous chelation irrigation protocol in endodontics: a scoping review of laboratory studies

**DOI:** 10.1007/s10266-023-00835-8

**Published:** 2023-07-11

**Authors:** Giusy Rita Maria La Rosa, Gianluca Plotino, Venkateshbabu Nagendrababu, Eugenio Pedullà

**Affiliations:** 1 Department of General Surgery and Medical-Surgical Specialties, University of Catania, Catania, Italy; 2 Private Practice, Rome, Italy; 3 Department of Preventive and Restorative Dentistry, College of Dental Medicine, University of Sharjah, Sharjah, UAE

**Keywords:** Continuous chelation, Endodontics, Etidronate, Scoping review, Sequential chelation

## Abstract

This scoping review aimed to synthesize and explore the current boundaries and limitations of laboratory research on the effectiveness of continuous chelation irrigation protocol in endodontics. This scoping review was reported according to the Preferred Reporting Items for Systematic Reviews and Meta-Analyses (PRISMA) Extension for Scoping Reviews. Literature search was conducted on Pubmed and Scopus to identify all laboratory studies evaluating smear layer and hard-tissue debris removal or, antimicrobial efficacy, or dentine erosion induced by continuous chelation. Two independent reviewers performed the all review steps and the relevant items were recorded. Seventy-seven potentially relevant studies were identified. Finally, 23 laboratory studies met the eligibility criteria for qualitative synthesis. Seven studies focused on the smear layer/debris removal outcome, 10 on antimicrobial activity, and 10 on dentine erosion. In general, the continuous chelation protocol was equally or more effective in the cleanliness of root canals and antimicrobial activity compared with traditional sequential protocol. In addition, etidronate solutions seemed to be milder chelating agents compared to those with EDTA, thus resulting in reduced or no dentine erosion and roughness modification. Yet, the methodological differences among the included studies limit the results’ generalizability. The continuous chelation seems to be equally or more effective in all investigated outcomes when compared with the traditional sequential protocol. The methodological variability among the studies and shortcomings in the methods employed limit the generalizability and clinical relevance of the results. Standardized laboratory conditions combined with reliable three-dimensional investigation approaches are necessary to obtain clinically informative findings.

## Introduction

The root canal treatment aims to eliminate the intracanal infection and avoid reinfection by obturation of the root canal space [1, 2]. Mechanical preparation is able to remove microorganisms from an infected root canal [3]. However, after instrumentation up to 35% of the canal surface area may remain unchanged [4], which may not guarantee a clean and bacteria-free root canal space. As a consequence, an active irrigation sequence is generally combined with the mechanical treatment [5]. Although the irrigants are crucial for the success of root canal treatment, it is important they do not damage the tissues surrounding the root, they are safe for both patient and clinician, and ensure full functional recovery of the tooth [6]. Sodium hypochlorite (NaOCl), a non-specific proteolytic agent available in different concentrations (0.5–6%), is used for its notable tissue solvent action, antimicrobial and anti-biofilm effects [7, 8]. However, NaOCl is unable to eliminate the smear layer and prevent the accumulation of hard-tissue debris [9]. Dentinal debris can act as a physical barrier that prevents NaOCl from reaching all anatomical anfractuosities [10, 11]. In addition, the presence of dentinal debris reduces the antimicrobial efficacy of NaOCl on dentinal structure [11]. Consequently, considering its inability to remove the inorganic tissue remnants, NaOCl is usually followed by a chelating agent such as ethylenediaminetetraacetic acid (EDTA), at a concentration of 15–17% for 1 − 2 min [12, 13].

Of note, chelating agents negatively impact the free available chlorine content of NaOCl and thus reduce its tissue dissolution ability, while the antimicrobial action decreases only when the initial NaOCl concentrations are low [14]. In order to prevent these phenomena, the sequential use of NaOCl/EDTA, known as the “sequential protocol”, is routinely used in day-to-day clinical practice. NaOCl is used as an antimicrobial agent during instrumentation, and EDTA is applied at the end of instrumentation to promote the smear layer removal [15]. A final flush of NaOCl has also been proposed to improve NaOCl penetration into the areas that were earlier covered with the smear layer [16].

The sequential protocol results in a wider opening of the dentinal tubules [17] and intertubular tunnelling due to dentine erosion [18]. NaOCl/EDTA determines the complete decalcification of the superficial 1 − 5 µm of intertubular dentine, and up to 20 µm of the dentinal tubular walls [17]. These structural changes significantly diminish the flexural strength of dentine [19–21] and may increase the risk of vertical root fractures [22].

To overcome the above issues caused by the sequential use of NaOCl/EDTA, the concept of “continuous chelation” was proposed in 2005. It refers to the combination of a soft chelator with NaOCl for simultaneous antimicrobial and proteolytic action with the smear layer removal [23–25]. According to this protocol, NaOCl is added with the salt of a weak chelator, 1 hydroxyethylidene-1, 1-bisphosphonate or etidronate (HEBP or HEDP or etidronate), because the tetra-sodium HEDP salt is extremely compatible with NaOCl [25]. Continuous chelation is an attractive concept because of its multiple benefits: simplification of the clinical procedure, improved debris removal [13], acceptable tolerability with some dental materials [26], and no reduction in NaOCl antimicrobial activity [25] and dissolving properties [23]. Moreover, chelators promote the detachment of biofilms from the root canal walls [27, 28] and eliminate the metal ions employed by bacteria as nutrients [29]. One of the major concerns associated with their application is the potential chemical reactions between NaOCl and the chelator. Indeed, NaOCl is able to chemically interact with other irrigants and the consequent mixing of two irrigants (i.e., chelators and antimicrobials) has different effects. It determines the pH reduction of the hypochlorite component and its decomposition to chlorine gas. In addition, the mixing generates intermediate toxic products able to reduce the clinical performance of NaOCl [30].

To date, the majority of available studies are performed in laboratory setting under different methodological conditions. To explore and define the current knowledge on the effectiveness of continuous chelation in endodontic research, a scoping review of current laboratory studies is appropriate. A scoping review is a flexible approach for exploring a broad question with the aim of synthesizing the existing knowledge boundaries, identifying the current gaps and addressing the future research [31]. The aim of this scoping review was to explore the current literature in relation to the effectiveness of continuous chelation compared to the sequential protocol in order to provide an overall and updated view for researchers to detect gaps and carry out further laboratory studies.

## Materials and methods

This scoping review was reported according to the Preferred Reporting Items for Systematic Reviews and Meta-Analyses (PRISMA) Extension for Scoping Reviews [32].

### Research question

This scoping review aimed to synthesize and explore the current boundaries and limitations of laboratory research on the effectiveness of continuous chelation irrigation protocol in smear layer and hard-tissue debris removal, antimicrobial efficacy and dentine erosion.

### Search strategy

A literature search was conducted in the PubMed and Scopus databases on 25 September, 2022 to identify all pertinent studies. The following search string was adopted for each database: (“continuous chelation” OR “soft chelation” OR “etidronate” OR “HEDP” OR “HEBP” OR “etidronic acid”) AND (“root canal irrigants” OR “irrigation” OR “antimicrobial efficacy” OR “smear layer” OR “debris” OR “dentine erosion”) AND (“endodontics”). No language restrictions were applied. Reference lists of included studies were further screened for other potential studies. Principal peer-reviewed scientific journals in endodontics (*Journal of Endodontics, International Endodontic Journal, Clinical Oral Investigations, Odontology and Australian Endodontic Journal*) were also hand searched. Two review authors independently reviewed and selected studies from searches. Disagreements were resolved through discussion or by the intervention of a third reviewer.

### Eligibility criteria

Laboratory studies evaluating smear layer and hard-tissue debris removal or antimicrobial efficacy or dentine erosion induced by continuous chelation compared to sequential chelation were included. The exclusion criteria included the study design (animal and human studies), outcome, comparator (i.e. no comparison with NaOCl and EDTA solutions), article type (editorials, commentaries, letters and reviews), peer-revision (abstracts and preprint articles) and language (studies without an English abstract).

### Data extraction

For each study, the following items:

- Name of first author
- Year of the study published
- Study design
- Sample size (n)
- Irrigants used
- Outcome(s)
- Measurement outcome(s)
- Main findings

were extracted and synthesized in study tables for each outcome investigated. Data were extracted independently by two reviewers. Any discrepancies were solved by discussion or help of a third reviewer.

## Results

**Fig. 1:**
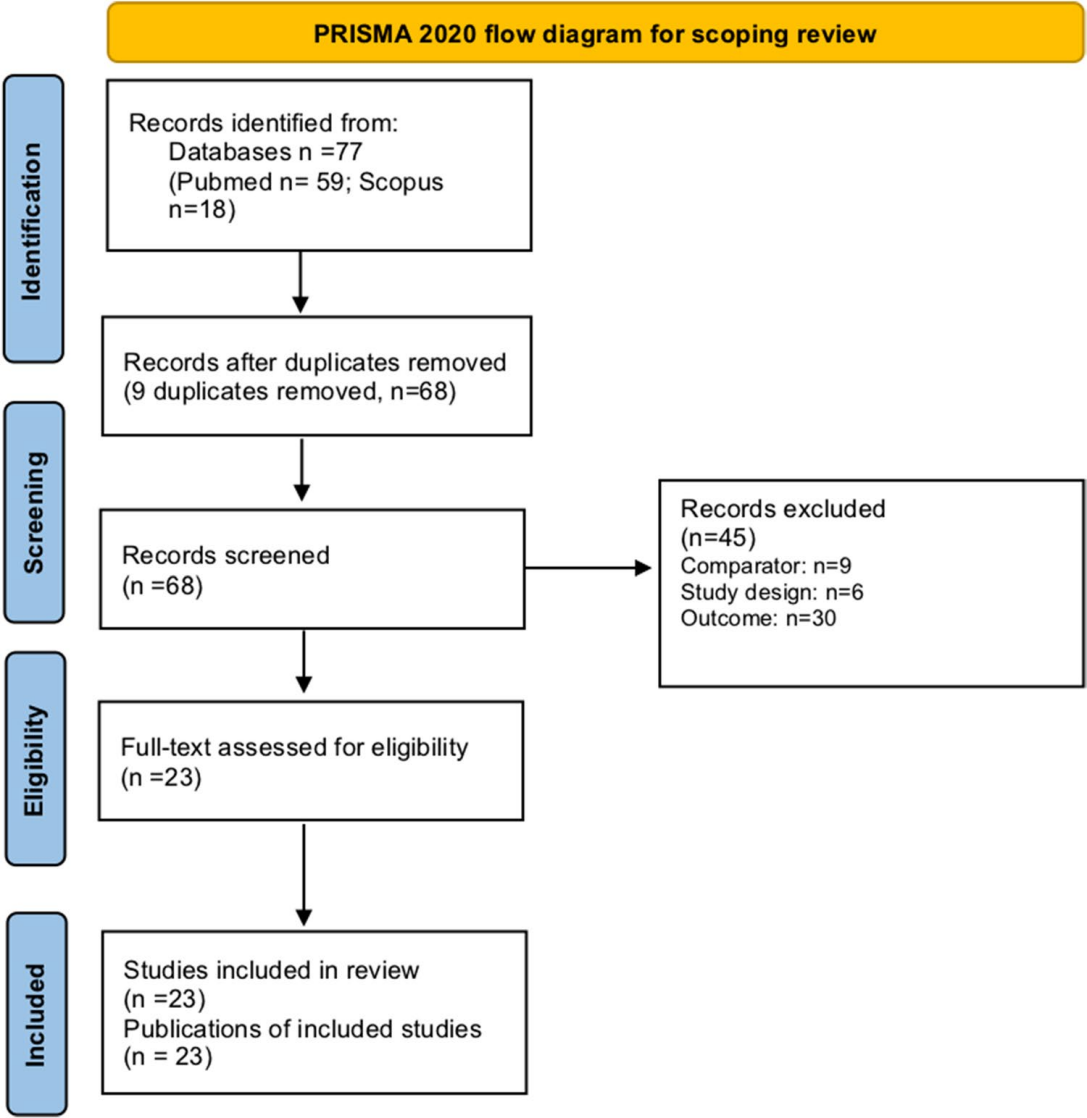
Flowchart of review process

Study selection is schematized in Fig. 1**,** according to PRISMA 2020 for scoping reviews. The search retrieved 77 potentially relevant studies. Duplicates (*n* = 9) and articles not satisfying the inclusion criteria (*n* = 45) were removed. Finally, 23 studies met the eligibility criteria for qualitative synthesis. The main features of the included studies are reported in Tables 1, 2, 3.

**Table 1:** General characteristics of the included studies for the smear layer/debris removal outcome

Author	Year	Study design	Sample size (*n*)	Irrigants used	Outcome(s)	Measurement outcome(s)	Main findings
Deari et al. [33]	2019	Blinded, randomized in vitro study on dentine disks of human third molars	10	12% Na_2_HEDP	Smear layer removal	Laser microscopy	Smear layer removal by EDTA and HEDP was affected by the pH values of the aqueous solution. EDTA was a stronger chelator than HEDP
				15% Na_4_HEDP			
				17% Na_2_EDTA			
				19% Na_4_EDTA			
				PBS (negative control group)			
Girard et al. [5]	2005	Blinded, randomized in vitro study on extracted single-rooted human premolars	16	Aqueous gel consisting of 2% alginate, 3% aerosil, 10% Tween 80 and 18% HEBP	Prevention of smear layer during root canal preparation	SEM	The examined HEBP gel had superior smear layer preventing ability compared with the paste-type chelator products containing EDTA and hydrogen peroxide available into the market
				Two commercial paste-type chelators containing EDTA and peroxide (RC-Prep and Glyde)			
				1% NaOCl without the use of a chelator (positive controls)			
Kfir et al. [34]	2020	Blinded, randomized in vitro study on extracted single-rooted human teeth	20	3% NaOCl + Dual Rinse HEDP	Cleanliness (amount of smear layer and debris)	SEM	Cleanliness of the two irrigant solutions was not significantly different
				3%NaOCl + 17%EDTA			
				Saline solution (negative control group)			
Lottanti et al. [17]	2009	Randomized in vitro study on extracted single-rooted human premolars	12	2% NaOCl + 18% HEBP	Smear layer removal	SEM	The tested decalcifying agents were all able to remove or prevent the smear layer formation
				1% NaOCl + 2.25% PAA			
				1% NaOCl + 17% EDTA [positive control]			
				1% NaOCl + water (negative control)			
Paqué et al. [13]	2012	Blinded, randomized in vitro study on extracted human mandibular molars	30	2.5% NaOCl + 9% HEBP	Hard-tissue debris removal	Micro-CT	The use of 2.5% NaOCl alone induced more significantly accumulated hard-tissue debris than 2.5% + 9% HEBP
				2.5% NaOCl + pure water			
Patil et al. [35]	2018	Blinded, randomized in vitro study on extracted single-rooted human mandibular premolars	10	Freshly mixed Chloroquick solution (18% etidronic acid + 5% NaOCl)	Smear layer removal in apical third of root canal	SEM	Sequential use of 5.25% NaOCl + 17% EDTA (both solution added with surfactant) was more efficient than MTAD and Chloroquick in the smear layer removal from the apical third
				5.25% NaOCl with surfactant + 17% EDTA with surfactant			
				Freshly mixed BioPure MTAD			
				Normal saline (negative control group)			
Ulusoy et al. [36]	2018	Randomized study on human extracted maxillary anterior teeth	12	2.5% NaOCl + 18%HEBP	Organic tissue removal	Weight by a precision balance	Activation by XP-endo Finisher caused the greatest weight tissue loss in the experimental solutions compared with PUI activation NaOCl + HEBP mixture plus XP-endo Finisher resulted an effective irrigation protocol for removing simulated organic tissue from artificial internal root resorptions cavities
				2.5% NaOCl			
				2.5% NaOCl + 17% EDTA			
				Distillated water			

*EDTA* ethylenediaminetetraacetic acid; *HEBP or HEDP* 1-hydroxyethylidene-1, 1-(Di)/(Bi)Sphosphonate, *Micro-CT* micro-computed tomography, *MTAD* mixture of doxycycline, citric acid and a detergent, *NaOCl* sodium hypochlorite, *PAA* peracetic acid; *PBS* phosphate-buffered saline, *PUI* passive ultrasonics irrigation, *SEM* scanning electron microscope

**Table 2:** General characteristics of the included studies for the antimicrobial activity outcome

Author	Year	Study design	Sample size [*n*]	Irrigants used	Outcome(s)	Measurement outcome[s]	Main findings
Arias-Moliz et al. [38]	2014	Randomized in vitro study on extracted human maxillary premolars	5	2.5% NaOCl + 9% HEBP	Antimicrobial activity on the growing of *Enterococcus faecalis* biofilms	CLSM and the live/dead technique	HEBP did not alter the ability of NaOCl to inhibit *E. faecalis* grown in biofilms and into the dentinal tubules
				2.5% NaOCl			
				9% HEBP			
				Distillated water (control group)			
Arias-Moliz et al. [39]	2015	Randomized in vitro study on extracted human molars	5	2.5% NaOCl + 9% HEBP	Antimicrobial effect on the growing of *Enterococcus faecalis* biofilms	CLSM and the live/dead technique	NaOCl alone or combined with HEBP was the most effective irrigant solution in disrupting and killing *E. faecalis* biofilms
				2.5% NaOCl			
				2% PAA			
				2% CHX			
				Distillated water (control group)			
Arias-Moliz et al. [27]	2016	Randomized in vitro study on dentine disks of extracted human teeth	5	1% NaOCl + 9% HEBP	Influence of dentine powder on the concentration, pH, and antimicrobial activity of irrigant solutions	CLSM and the live/dead technique	Dentine powder negatively impacted the available chlorine and antimicrobial activity of 1% NaOCl, 2.5% NaOCl, and 1% NaOCl/HEBP; yet, it did not influence the antimicrobial activity of 2.5% NaOCl/HEBP after a 3-min contact time against *E. faecalis* biofilms
				2.5% NaOCl + 9% HEBP			
				1% NaOCl			
				2.5% NaOCl			
				9% HEBP			
				Distillated water (control group)			
Borges et al. [44]	2022	In vitro study on dentine discs of extracted human teeth	9	2% and 5% NaOCl + HEDP	Antibiofilm potency (biofilm removal and disruption, rate of biofilm loss and disruption, bubble formation) against dual-species biofilm (*Streptococcus oralis J22* and *Actinomyces naeslundii T14V-J1*)	OCT	Higher NaOCl concentrations were associated with a major biofilm removal and disruption and bubble formation. The HEDP delayed the anti-biofilm action of NaOCl but not reduced its antimicrobial efficacy
				2% and 5% NaOCl			
Campello et al. [40]	2022	Randomized in vitro study on extracted human mandibular premolars	21	2.5%NaOCl/9%HEDP	Antibacterial activity against *Enterococcus faecalis*	Quantitative RPCR	Both the freshly combined NaOCl/HEDP and the alternate use of NaOCl/ CA, activated by XP-endo Finisher, significantly diminished bacterial count compared with NaOCl alone Under no activation, NaOCl/HEDP was significantly more effective than the two other techniques
				2.5%NaOCl			
				2.5%NaOCl/10% CA			
Giardino et al. [37]	2019	Blinded, randomized in vitro study on extracted single-rooted human teeth	5 for surface tension test 10 for percentage of viable bacteria	5%NaOCl + Dual Rinse HEDP	The surface tension and the antimicrobial activity of irrigant solutions against E. faecalis	Wilhelmy plate technique; CLSM and the live/dead technique	Dual Rinse HEDP increased NaOCl antimicrobial effect in dentinal tubules even if enhanced its surface tension
				5%NaOCl			
				17% EDTA			
Morago et al. [41]	2016	Randomized in vitro study on extracted human premolars	5	2.5% NaOCl + 9% HEBP	The influence of the smear layer on the antimicrobial activity of irrigant solutions	CLSM and the live/dead technique	With no smear layer, 2.5% NaOCl alone and plus 9% HEBP showed high antimicrobial activity with no differences between them The smear layer reduced the antimicrobial activity of 2.5% NaOCl while did not affect the antimicrobial capacity of 2.5% NaOCl/9% HEBP
				2.5% NaOCl			
				Distillated water (control group)			
Neelakantan et al. [42]	2015	Randomized in vitro study on extracted single-rooted human premolars	80 per group; 20 per subgroup	6% NaOCl + 18% HEDP	The impact of three irrigation protocols, activated by three different methods, on mature biofilms of *Enterococcus faecalis*	CLSM and the live/dead technique	No significant differences emerged between NaOCl + etidronic acid and NaOCl-EDTA-NaOCl, whereas both groups induced more bacterial reduction than NaOCl-EDTA Diode laser and Er: YAG laser activation were superior compared with ultrasonics in the dentinal tubule disinfection
				3% NaOCl + 17% EDTA			
				3% NaOCl + 17% EDTA + 3% NaOCl			
				Sterile saline (control group)			
Pedrinha et al. [43]	2021	Randomized in vitro study on extracted human lower incisors	10	5% NaOCl + 18% HEBP	Canal and intratubular decontamination against *Enterococcus faecalis*	CFU/mL count; CLSM and the live/dead technique	NaOCl + EDTA-T had the best intratubular antibacterial activity, mainly when associated with XP-Endo Finisher activation
				2.5% NaOCl + 17% EDTA			
				2.5% NaOCl + EDTA-T			
Zehnder et al. [25]	2005	Blinded, randomized in vitro study on extracted single-rooted human teeth	6	1% NaOCl + 7% HEBP	The interactions of chelators with NaOCl solution [antibacterial efficacy and smear layer]	Standard iodine/thiosulfate titration method; SEM; atomic absorption spectrometry	NaOCl did not modify the calcium-complexing ability of chelators; EDTA and CA negatively interfered with NaOCl antimicrobial activity, while HEBP did not
				1% NaOCl/H_2_O;			
				7% HEBP/H_2_O			
				17% EDTA/H_2_O			
				17% EDTA/1% NaOCl			
				10% CA/H_2_O			
				10% CA/1% NaOCl			

*CA* citric acid, *CFU* colony-forming unit, *CLSM* confocal laser scanning microscopy, *CHX* chlorhexidine, *EDTA* ethylenediaminetetraacetic acid, *EDTA-T* EDTA plus sodium lauryl ether sulfate, *H*_*2*_*O* hydrogen peroxide, *HEBP or HEDP* 1-Hydroxyethylidene-1, 1-(Di)/(Bi)Sphosphonate, *NaOCl* sodium hypochlorite, *OCT* optical coherence tomography, *PAA* peracetic acid, *RPCR* real-time polymerase chain reaction, *SEM* scanning electron microscope, *Er:YAG* pulsed erbium:yttrium–aluminum-garnet laser

**Table 3:** General characteristics of the included studies for the dentine erosion/roughness outcome

Author	Year	Study design	Sample size [*n*]	Irrigants used	Outcome(s)	Measurement outcome[s]	Main findings
Deari et al. [33]	2019	Blinded, randomized in vitro study on dentine disks of extracted human third molars	10	12% Na_2_HEDP	Dentine decalcification	ABS	Dentine decalcification by EDTA and HEDP depended on pH values of the aqueous solution; EDTA was a more potent calcium sequestrant than HEDP
				15% Na_4_HEDP			
				17% Na_2_EDTA			
				19% Na_4_EDTA			
				PBS (negative control group)			
Dineshkumar et al. [45]	2012	Randomized in vitro study on extracted single-rooted human mandibular premolars	20	1.3% NaOCl and 18% HEBP	Dentine microhardness	Vickers microhardness test	Among the tested solutions, HEBP reported the highest dentine microhardness; MTAD the least. HEBP as a final rinse seemed to less impact the mineral content of root dentine
				1.3% NaOCl + 17% EDTA			
				1.3% NaOCl + MTAD			
				Distilled water			
Girard et al. [3]	2005	Blinded, randomized in vitro study on extracted single-rooted human premolars	16	Aqueous gel consisting of 2% alginate, 3% aerosil, 10% Tween 80 and 18% HEBP	Calcium chelating capacity	Calcium-selective measuring chain	The examined HEBP gel had higher calcium chelating capacity compared with paste-type chelator products containing EDTA and hydrogen peroxide available into the market
				Two commercial paste-type chelators containing EDTA and peroxide (RC-Prep and Glyde)			
				1% NaOCl without the use of a chelator [positive controls]			
Kfir et al. [34]	2020	Blinded, randomized in vitro study on extracted single-rooted human teeth	20	3% NaOCl + Dual Rinse HEDP	Erosion of root canal walls	SEM	The two tested irrigant solutions did not differ for dentine erosion
				3%NaOCl + 17%EDTA			
				Saline solution (negative control group)			
Lottanti et al. [17]	2009	Randomized in vitro study on extracted single-rooted human premolars	12	2% NaOCl + 18% HEBP	Calcium eluted from the canal system and root dentinee demineralization	ABS; SEM	The tested decalcifying agents eroded the dentine wall in different manner. NaOCl/ etidronic acid did not decalcify the canal walls when used during root canal instrumentation and in the final rinse
				1% NaOCl + 2.25% PAA			
				1% NaOCl + 17% EDTA [positive control]			
				1% NaOCl + water (negative control)			
Rath et al. [47]	2020	Randomized in vitro study on extracted single-rooted human mandibular premolars	12	3% NaOCl + 18% HEDP	Ultrastructural matrix characteristics and the chemical composition of dentine	SLM; STM; FTIR and EDS; ninhydrin assay	NaOCl/HEDP caused partially degraded, yet mineralized collagen fibers while NaOCl/EDTA dissolved the hydroxyapatite encapsulation, with the collagen fibre bundles exposition; NaOCl/HEDP showed a uniform distribution of organic and inorganic elements
				3%NaOCl + 17%EDTA			
				Normal saline solution (control group)			
Tartari et al. [46]	2013	Randomized in vitro study on extracted human anterior teeth	9	5% NaOCl + 18% HEBP	Root dentine roughness	Profilometer	NaOCl did not impact the surface roughness; only the irrigation protocols involving chelating agents altered the roughness of root dentine
				2.5% NaOCl + 9% HEBP			
				2.5% NaOCl + 9% HEBP + 2.5% NaOCl			
				2.5% NaOCl + 17% EDTA			
				2.5% NaOCl + 10% CA			
				2.5% NaOCl + 17% EDTA + 2.5% NaOCl			
				2.5% NaOCl + 10% CA + 2.5% NaOCl			
				Saline solution [control]; 2.5% NaOCl			
Tartari et al. [48]	2017	In vitro study on dentine slices of bovine incisors	5	9% and 18% HEDP	Dentine demineralization	ATR-FTIR	EDP and EDTANa_4_ determined minor while EDTAHNa3 and PAA greater demineralization of dentine. Both effects were time and concentration dependent. NaOCl degraded the dentine organic matrix more quickly when the matrix was exposed
				0.9% saline			
				5% and 10% EDTANa_4_;			
				17% EDTAHNa_3_			
				0.5% and 2.0% PAA			
				The combination of the previous agents with NaOCl			
Tartari et al. [49]	2018	In vitro study on dentine slices of bovine incisors	10	2.5% NaOCl + 9% HEDP	Dentine roughness	Roughness measuring station	Saline solution, NaOCl, HEDP and CHX did not change the roughness of the dentine, whilst EDTA and PAA did
				2.5% NaOCl + 9%HEDP + 2%CHX			
				mixture of 5% NaOCl + 18% HEDP			
				2.5% NaOCl			
				2.5% NaOCl + 17% EDTA			
				2.5% NaOCl + 0.5% PAA			
				2.5%NaOCl + 17%EDTA + 2%CHX			
				2.5% NaOCl + 0.5%PAA + 2%CH			
				0.9% saline solution			
Ulusoy et al. [50]	2020	In vitro study on extracted single-rooted human mandibular teeth	10	9% HEBP	Dentine nanohardness and erosion	Nanoindenter; SEM	Final irrigation with etidronate alone or combined with NaOCl altered structurally the root canal dentine Single chelator and chelator plus NaOCl had no significantly impact on dentine nanohardness
				2.5% NaOCl + 9% HEBP			
				2.5% NaOCl			
				17% EDTA			
				2.5% NaOCl + 17% EDTA			
				2% PAA			
				2.5% NaOCl-2% PAA			
				Distilled water (control)			

*ABS* atomic absorption spectroscopy, *ATR-FTIR* attenuated total reflectance in fourier transform infrared spectroscopy, *CA* citric acid, *CHX* Chlorhexidine, *EDS* energy-dispersive-x-ray spectroscopy, *EDTA* ethylenediaminetetraacetic acid, *FTIR* fourier transform infrared spectroscopy, *HEBP Or HEDP* 1-hydroxyethylidene-1, 1-(Di)/(Bi)sphosphonate, *MTAD* mixture of doxycycline, citric acid and a detergent, *NaOCl* sodium hypochlorite, *PAA* peracetic acid, *PBS* phosphate-buffered saline, *SEM* scanning electron microscope, *SLM* scanning light microscopy, *STM* scanning transmission microscopy

### Smear layer/debris removal

The studies retrieved for smear layer/debris removal outcomes are shown in Table 1. Five blinded, randomized studies on extracted human teeth [3, 13, 33–35] were identified. Two studies were randomized but no blinding was reported [17, 36]. Four studies tested HEBP (9–18%) combined with different NaOCl concentrations (2–3%) [13, 17, 34, 36], one study in an aqueous gel consisting in 2% alginate, 3% aerosil, 10% Tween 80 [3] and one with EDTA to obtain Na_2_ and Na_4_ salts of HEDP [33]. One study tested the efficacy of two techniques for activation of irrigants (i.e., XP-Endo Finisher and passive ultrasonics irrigation) [36]. Different testing methods were employed to determine the smear layer/debris removal including a precision balance [36], scanning electron microscopy (SEM) [3, 17, 34, 35], laser microscopy [33] and micro-CT [13].

Overall, the continuous chelation protocol was equally or more effective in the cleanliness of root canals (smear layer/debris removal) when compared with the traditional sequential protocol [3, 13, 17, 34, 36]. Patil et al*.* [35] reported that sequential use of 5.25% NaOCl + 17% EDTA (both combined with surfactants) was more efficient than 18% etidronic acid + 5% NaOCl in the removal of smear layer in the apical third. According to Deari et al*.* [33], EDTA was a stronger chelator than HEDP. Nevertheless, none of the chelating solutions was able to completely remove smear layer and debris from the root canal walls.

### Antimicrobial activity

Table 2 reports the details of the ten studies identified for the antimicrobial activity outcome. Two blinded, randomized studies on extracted human teeth [25, 37], seven randomized with no reporting on blinding procedures [27, 38–43] and one with no information on randomization and blinding [44] were retrieved. All studies tested HEBP (5–18%) combined with different NaOCl concentrations (1–6%). Three studies tested the efficacy of techniques for activation of irrigants (i.e., XP-Endo Finisher, diode laser, Er: YAG laser activation, passive ultrasonics irrigation) [40, 42, 43]. Antimicrobial activity was principally assessed by confocal laser scanning microscopy [27, 37–39, 41–43]. Other testing methods include optical coherence tomography [44], quantitative real-time polymerase chain reaction [40], SEM and atomic absorption spectrometry [25].

In general, the continuous chelation protocol was equally or more effective in antimicrobial activity when compared with the traditional sequential protocol [25, 27, 37–42]. Pedrinha et al*.* [43] reported that NaOCl + EDTA-T (i.e., EDTA plus sodium lauryl ether sulfate) showed the best intratubular antibacterial activity. Furthermore, the addition of HEBP delayed the anti-biofilm action of NaOCl but did not compromise its antimicrobial efficacy [44]. However, none of the solutions were able to completely eliminate bacteria from the root canals.

### Dentine erosion

Table 3 lists the studies retrieved for dentine erosion outcome. Three of ten selected laboratory studies were blinded and randomized on extracted human teeth [3, 33, 34], four did not report blinding procedures for examiners [17, 45–47] and three did not specify blinding or randomization techniques [48–50]. Two studies tested HEBP (9–18%) alone [48, 50], eight combined with different NaOCl concentrations (1.3–5%) [17, 34, 45–50], one with NaOCl-Chlorhexidine [49], one in an aqueous gel consisting of 2% alginate, 3% aerosil, 10% Tween 80 [3] and one with EDTA to obtain Na_2_ and Na_4_ salts of HEDP [33].

Testing methods to assess the dentine erosion and roughness modification were varied and included nanoindenter [50], Vickers microhardness test [45], SEM [17, 34, 50], atomic absorption spectroscopy [17, 33], attenuated total reflectance in Fourier-transform infrared spectroscopy [48], scanning light and transmission microscopies [47], Fourier-transform infrared spectroscopy [47], energy-dispersive-X-ray spectroscopy [47], profilometer [46] and roughness measuring station [46].

Overall, HEDP liquid irrigant solutions seemed to be milder chelating agents compared to those with EDTA, thus resulting in reduced or no dentine erosion and roughness modification when the continuous chelation protocol was used [17, 33, 34, 45, 47–49]. Conversely, Ulusoy et al*.* [50] reported that final irrigation with etidronic acid alone or in association with NaOCl altered structurally the root canal dentine. According to Girard et al*.* [3], the tested HEBP gel demonstrated major calcium chelating capacity compared with the marketed paste-type chelator products containing EDTA and hydrogen peroxide. Finally, Tartari et al*.* [46] showed that NaOCl did not influence the surface roughness; only the irrigation protocols including chelating agents modified the roughness of root dentine.

## Discussion

### Smear layer and debris removal

Mechanical instrumentation of the root canal is likely to produce hard debris [13] and inorganic shavings, generating a smear layer [51, 52] that covers the walls of the prepared root canal [53, 54]. More specifically, smear layer was defined as “a layer of material composed of dentine, remnants of pulp tissue and odontoblastic processes, and sometimes bacteria” [55] and debris may contain “pulp tissue fragments, necrotic tissue, microorganisms, dentine chips and canal irrigants” [56]. Accumulated hard-tissue debris is clinically unfavourable, because it is conceivable that microorganisms remaining in anatomical ramifications after instrumentation may be protected by debris from disinfectants that are used in the main root canal system [13]. Moreover, accumulated debris might negatively affect the canal sealability [57] and interfere with NaOCl antimicrobial activity, reducing its effectiveness due the inability of the NaOCl solution to dissolve the smear layer [41]. This mechanism could be explained by the function barrier exerted by smear layer and the accelerated consumption of free available chlorine mediated by the organic components [27, 58, 59]. NaOCl cannot dissolve inorganic smear layer components [9]. Hence, the use of calcium-complexing (chelating) agents is suggested. They can be applied in a liquid or past-type formulation [9]. Several studies evaluated the efficacy of HEBP as an alternative to traditional chelating agents such as EDTA. Evidence from the included studies is limited by the extreme variability in terms of concentration of the NaOCl irrigant solutions, the irrigant formulation (i.e., liquid and paste-type), the type and concentration of HEBP, the time of irrigant application and the measurement of outcome. All included studies were randomized and conducted on extracted teeth or samples of teeth. Only one study [17] did not specify whether it was blinded; the others were blinded.

According to Kfir et al*.* [34] and Lottanti [17], cleanliness of HEDP-based irrigating solutions was not significantly different from NaOCl, followed by EDTA [17, 34]. Interestingly, the apical part did not present debris [34], in contrast with some previous studies which showed the apical part was associated with a notable quantity of debris when a syringe and needle were used for an irrigation procedure [60, 61]. Kfir et al*.* [34] suggest that this difference could be due to the small dimensions of the irrigation needle used (i.e., 30 G) which ensured to achieve the last 1–2 mm from the root canal apex, resulting in an effective cleanliness of the apical part. Furthermore, these contrasting findings could also due to a methodological systematic error occurred in previous studies, as suggested by Lottanti et al*.* [17]. Indeed, conventional investigations assessed the amount of smear layer on root canal walls evaluating exclusively the number of open tubules in a limited canal wall area. However, tubular sclerosis is most accentuated in the apical zona [62] and consequently, this physiological condition could have affected the smear layer evaluation in this anatomical area.

As reported by Deari et al*.* [33], smear layer removal by chelating agents such as EDTA and HEDP could be affected by pH values. More specifically, the sodium ions present in tetra-sodium salts induced an alkaline pH in solution. This caused a reduction of the decalcifying action of both molecules under investigation (i.e., EDTA and HEDP) in comparison with their disodium preparations. Moreover, they stated that EDTA was a stronger chelator than HEDP.

Patil et al*.* [35] reported that sequential use of 5.25% NaOCl with surfactant + 17% EDTA with surfactant was more efficient than a mixture of Doxycycline/citric acid/detergent and of Chloroquick (etidronic acid based-solution) in the smear layer elimination from the apical third. A direct comparison with other included studies is challenging because of the differences in methodological procedures, including the agent formulation, the use of surfactant, the sample, and the procedure overall. According to Girard et al*.* [3], the HEBP gel exhibited higher hypochlorite compatibility, calcium chelating ability, and smear layer preventing action when compared with paste-type chelator products with EDTA and hydrogen peroxide available into the market. Nevertheless, one possible limit of paste-type chelators use is the difficulty in achieving a smear-free apical root canal portion [9]. Rotating instruments can move the chelator away from the apical area, reducing the action of the calcium chelating agent in that area. Therefore, an additional chelating solution delivered by a fine needle should be used to reach the apical zone at the end of shaping [3].

When comparing different studies on smear layer removal by endodontic irrigants, methodological pitfalls frequently affect the results obtained [63]. Studies on dentine surface topography frequently use SEM [34]. Scanning electron microscope analysis of root canal walls has been a matter of extensive discussion in the late 2000s. Obvious biases were identified and discussed previously [63, 64]. The smear layer is a phenomenon that depends on dentine instrumentation. Thus, it is difficult to know if the observed areas after treatment were smear layer free before the SEM analysis [63]. It assesses a small area of the root canal that may be not representative of the entire surface. Additionally, it is commonly unknown how such areas are chosen during evaluation or whether the operator is blinded to them (i.e., operator bias) [65]. Moreover, quantifying smear layer presence can be complicated by the extent of sclerotic dentin in samples [62]. Finally, issues arise when drying and coating specimens since these processes can introduce various artefacts [63].

Overall, the results by score-based conventional SEM studies are not trustworthy and reproducible [63]. A valid alternative could be the 3D laser scanning microscope. It allows a simplified sample management at environmental conditions, ensuring high-quality images [33]. Micro-CT could be considered a valid alternative for assessing hard debris reduction[13]. Finally, it is important to emphasize that any type of irrigant cannot guarantee total cleaning of the root space and additional methods of cleaning should be considered [34]. Activation methods of the irrigant, such as mechanical scrubbing [66] or XP-Finisher (FKG Dentaire, La Chaux de-Fonds, Switzerland) [67] may influence the removal of debris and smear layer mainly from the apical root canal [34]. These laboratory-based results do not allow definitive conclusions to be reached about the substances tested. Indeed, clinical performance can be affected by multiple factors, such as the presence of blood and tissue remnants [3].

Within the methodological and procedural differences between the included studies, etidronate-based solutions and the continuous chelation protocol seem to be equally or more effective in the cleanliness of root canals when compared with traditional chelating agents and the sequential protocol. Yet, the 2D methodologies employed limit the clinical reliability of these results.

### Antimicrobial efficacy

Dentine infection is linked with pulp necrosis and the presence of biofilms in the root canal space. The complexity of the anatomical space prevents mechanical instrumentation alone from removing adherent biofilm [68]. Sodium hypochlorite is widely used for the removal of biofilm in the root canal space. The antimicrobial efficacy of NaOCl is dependent on its free chlorine form, which is influenced by several factors such as concentration, exposure time, pH, temperature, interaction with other organic or inorganic substances, or interaction with chelating agents [25, 59]. Combining an oxidizing agent (e.g., NaOCl) with a chelating agent (e.g., EDTA or citric acid) causes a chemical interaction and exothermic reaction [69]. The reaction reduces the amount of chlorine in NaOCl solutions, which makes the solutions less antimicrobial and less able to dissolve pulp [37].

Although differences in the methodology, substrate and volumes of NaOCl make comparison difficult, all included studies demonstrated that HEBP did not interfere with the antimicrobial ability of NaOCl [25, 38, 39, 42, 44] and under certain circumstances, may significantly increase the bacterial reduction [27, 37, 40, 41]. Indeed, the continuous chelation protocol penetrated deeper into the bacterial biofilm matrix, disrupting it and exposing the bacteria to NaOCl action [42]. Interestingly, according to Neelakantan et al*.* [42], NaOCl plus etidronic acid or NaOCl-EDTA-NaOCl guaranteed better dentinal tubule disinfection than NaOCl-EDTA. Consequently, the application of a disinfecting solution (e.g., NaOCl) after EDTA and continuous chelation caused significantly higher bacterial reduction.

Furthermore, some studies investigated the influence of smear layer or dentine powder on the antimicrobial activity of NaOCl alone or combined with HEBP [27, 37, 41]. Interestingly, in the study of Arias-Moliz et al*.* [27], a significantly higher antimicrobial activity emerged in the 1% HEBP solution compared with the solution without the chelator. This phenomenon could be due to the interaction of HEBP with biofilm structure and the inorganic components of infected dentine [17] which causes the bacteria detachment from the dentine surface also with sub-lethal chlorine concentration [27]. In addition, the mixing of HEBP and 2.5% NaOCl prevented NaOCl inactivation by dentine when dentine powder was present. These results are in agreement with those obtained by Morago et al*.* [41] who reported that the antimicrobial activity of 9% HEBP–2.5% NaOCl was not impacted by the smear layer. The higher activity of the combination of NaOCl with HEBP rather than NaOCl alone may be associated with the HEBP ability to remove the smear layer [17], probably allowing NaOCl to penetrate better into the dentine structure to exert its bactericidal action [41]. When the smear layer is present, the limited bactericidal action of NaOCl in all concentrations may be related to its inability to dissolve the smear layer [11]. This phenomenon has multiple explanations. First, the interaction between NaOCl and organic components of the smear layer could accelerate the consumption of the available free chlorine, thus deactivating the solution [27, 59]. Second, the smear layer may act as a barrier, preventing the irrigant from reaching infected dentinal tubules [41].

Nevertheless, according to Giardino et al*.* [37], adding a compatible chelator (i.e., corresponding to Dual Rinse HEDP in that study) to a NaOCl solution may increase its surface tension. The high surface tension could represent an obstacle for irrigating solution in achieving the root canal space for an extensive cleaning [37]. Conversely, the NaOCl/Dual Rinse HEDP mixture exhibited a better antibacterial action than NaOCl + EDTA. This result could be explained by the fact that this weak chelator, once dissolved in NaOCl, did not alter the antimicrobial action of NaOCl with no significant reduction of chlorine available within the first 60 min [25]. Moreover, the combination of etidronate powder with NaOCl makes the solution hypertonic and could intensify the antimicrobial efficacy by means of an osmotic effect. Indeed, hypertonic salt solutions are able to promote bacterial cell death and diminish the cohesion of biofilm matrices [70, 71].

According to Pedrinha et al*.* [43], NaOCl + EDTA-T showed the best intratubular antibacterial activity, particularly associated with XP-Endo Finisher activation, when compared with 5% NaOCl + 18% HEBP. The main methodological difference with the previous study is the application of activation techniques for irrigants. Mechanical devices and sonic, ultrasonic, and lasers techniques have been proposed as additional methods to increase the antibacterial and anti-biofilm activity of root canal irrigants, including etidronate [42, 72–75]. Nevertheless, the efficacy evaluation of the above tools was beyond the scope of the present review.

Generally, studies on the antimicrobial efficacy of irrigant solutions employ a single-species culture of *E. faecalis* [37, 41]. Although endodontic infections are polymicrobial [41, 76], this bacterial strain is widely selected in laboratory studies, because most endodontic retreatments were found to be caused by *Enterococcus faecalis* [77, 78]. The culture-based method has been considered the gold standard to evaluate the residual infection in the root canal space [79]. Anyway, assessing the antimicrobial activity of an irrigant on dentine substrate presents some limitations due to the culture methods employed [38]. Most of the included studies on the antimicrobial activity of irrigating solutions used Confocal Laser Scanning Microscope (CLSM) analysis. In contrast to traditional culture methods, CLSM analysis makes it possible to assess the proportion of dead/living bacteria without interfering with the cells attached to the substrate [39, 80, 81].

It is pivotal to underline that root canal anatomy is complex and direct applications of laboratory results in clinical practice require prudence [25, 42]. Despite these limitations, based on the results of the included studies, the continuous chelation protocol seems to be equally or more effective in antimicrobial activity when compared with the traditional sequential protocol.

### Dentine erosion

Chelator-induced erosion of the root canal walls negatively affects the mechanical properties of dentine [20, 82, 83]. Most of the included studies confirmed that HEDP was milder than EDTA in inducing dentine demineralization [17, 33, 45, 47, 48]. Conversely, Ulusoy et al*.* [50] reported that final irrigation with etidronic acid alone or in association with NaOCl altered structurally the root canal dentine compared with a single chelator and a chelator combined with NaOCl. These findings are probably related to the methodological procedure, including exposure time, irrigating protocol, and outcome measure. Studying the root canal appearance by images, indeed, could be misleading because of the notable heterogeneity of root dentine [17]. More sensitive alternatives to investigate directly and indirectly how irrigants modify the composition of dentine are digital techniques such as energy-dispersive X-ray spectroscopy (EDS) [84, 85], atomic force microscopic imaging (AFM) [84], microhardness and roughness tests [86] and Attenuated Total Reflectance in Fourier Transform Infrared Spectroscopy (ATR-FTIR) [87]. The considerations previously mentioned on limits of SEM are equally valid for dentin erosion investigations [63].

A study comparing the chelating ability of gel solutions containing HEDP or EDTA reported that the HEBP had superior calcium chelating capacity as opposed to the available paste-type chelator products constituted by EDTA and hydrogen peroxide [3]. Also, in this situation, it is complex to compare these results with those of previous studies due to the methodological differences such as composition and type of irrigant tested (i.e., gel vs liquid). As observed for smear layer removal, calcium chelation seems to be affected by pH solution [33]. More specifically, considering the di-and tetra-sodium salts, the high quantity of metal ions in the tetra-sodium formulation may impact the chelation activity of the sequestrant, with less chelation for Na_4_. Of note, the disodium salt solutions exhibited a lower pH compared to the tetra-sodium ones, which could have influenced the calcium dissolution from dentine discs. These results are in agreement with previously published data [48, 49]. In addition, EDTA and HEDP chelators induced different patterns of dentine decalcification. As reported by Rath et al*.* [47], dentine surface exposed to the NaOCl/EDTA protocol exhibited “naked” collagen fibers that were free of mineral encapsulation. On the other hand, the NaOCl/HEDP protocol presented a surface erosion and disorientation of the organic matrix at the interface. However, the collagen fibrils were embedded by minerals and a uniform structure of organic and inorganic elements was still appreciable [47]. Such limited alterations of dentine composition with no modifications in the amide and phosphate ratio have been previously reported [49, 88].

The collagen microstructure has a pivotal role in determining the biomechanical properties of dentine. More in detail, the fracture toughness of dentine depends on collagen and water content [89, 90]. Consequently, any procedure that alters the fibrillary arrangement of dentine matrix negatively impacted the flexural strength, potentially favouring the dentine fracture [91, 92]. Moreover, it is expected that the exposed collagen fibers have to be encapsulated by root canal sealers such as epoxy resins [93]. Anyway, exposed collagen fibers free of mineral protection or not embedded by root canal fillings are susceptible to bacteria-derived proteolytic enzymes [94], contributing to clinical failure [34]. Changes occurring in dentine structure may also influence the surface roughness of dentine tissue [46]. A limited enhancement in surface roughness could be clinically auspicious, because it may increase the micromechanical bonding of root canal sealers [86, 95]. Nevertheless, accentuated roughness can promote bacterial colonization [96]. According to Tartari et al*.* [49], HEDP did not alter the dentine roughness, whereas EDTA did. Kfir et al*.* [34] reported the HEDP-based irrigation solution was not significantly different from 3% NaOCl + EDTA in causing erosion of the canal wall. Conversely, as reported by Tartari et al*.* [46], the regimens that employed citric acid and HEBP combined with NaOCl showed a greater increase in roughness than other groups, including those containing EDTA. The different findings are probably related to the methodological conditions, including the irrigation protocol, application time, irrigant concentration, and the technique of analysis used for measuring dentine hardness.

Within the methodological differences between the included studies, on the basis of the current knowledge, HEDP irrigant seems to be a milder chelating agent compared with EDTA, thus resulting in reduced or no dentine erosion and roughness modification when the continuous chelation protocol was used. However, methodological limitations hinder the reliability of results similarly to debris removal outcome.

Scoping review guarantees a more flexible methodological approach in which the quality assessment of included studies is not mandatory, because the primary aim is to explore a broad topic for identifying the state of current knowledge, hypothetical gaps, and directing the future research [31]. For all these reasons, a scoping approach instead of a systematic was chosen. Moreover, considering the notable variety in laboratory conditions among the included studies, a meta-analysis was not conducted. Furthermore, the effect of continuous chelation on the bond strength of endodontic sealers, root transportation, and fracture resistance has not been assessed in the current review and requires further investigation.

In addition, the included studies presented notable differences regarding the type of samples, the irrigation protocol, the application time and concentration of irrigant solution, the outcome and measurement. Hence, a comparison is challenging, and the findings should be interpreted with caution. Moreover, the above-mentioned limitations of two-dimensional investigation methods prevent from obtaining reliable and trustworthy results especially for smear layer/debris removal and dentin erosion outcomes. Future studies should be based on three-dimensional techniques which allow a straightforward, standardized and not operator-dependent sample analysis.

Despite the standardized conditions, laboratory studies cannot fully reproduce the complexity of oral conditions, including pH, dentine structure and ageing and root canal microbiota. When the outcome allows it, high-quality randomized clinical trials should be preferred.

## Conclusions

Overall, most of the included studies showed that continuous chelation seems to be equally or more effective in smear layer/debris removal, antimicrobial activity, and dentine erosion when compared with the traditional sequential protocol. Yet, included studies differ among each other in terms of samples, irrigation protocol, application time and concentration of irrigant solution and outcome measure making comparison difficult. Moreover, investigation methods applied in the current research are often inadequate (i.e., SEM). For future laboratory-based studies to be more informative, they should use a standardized and comparable experimental protocol with reliable and unbiased investigation methods.

## Data Availability

Data sharing not applicable—no new data generated, or the article describes entirely theoretical research.
